# Validation of the Substance Use Risk Profile Scale (SURPS) With Bulgarian Substance Dependent Individuals

**DOI:** 10.3389/fpsyg.2018.02296

**Published:** 2018-11-26

**Authors:** Elizabeth C. Long, Svetla Milcheva, Elena Psederska, Georgi Vasilev, Kiril Bozgunov, Dimitar Nedelchev, Nathan A. Gillespie, Jasmin Vassileva

**Affiliations:** ^1^Institute for Drug and Alcohol Studies, Virginia Commonwealth University, Richmond, VA, United States; ^2^Department of Psychiatry, University Hospital Sveta Marina, Varna, Bulgaria; ^3^Bulgarian Addictions Institute, Sofia, Bulgaria; ^4^Department of Cognitive Science and Psychology, New Bulgarian University, Sofia, Bulgaria; ^5^Virginia Institute for Psychiatric and Behavioral Genetics, Virginia Commonwealth University, Richmond, VA, United States; ^6^Department of Genetic Epidemiology, QIMR Berghofer Medical Research Institute, Brisbane, QLD, Australia; ^7^Department of Psychiatry, Virginia Commonwealth University, Richmond, VA, United States

**Keywords:** heroin dependence, amphetamine dependence, Bulgaria, psychometrics, factor analysis

## Abstract

**Background:** The Substance Use Risk Profile Scale (SURPS) is a 23-item self-report questionnaire that assesses four well-validated personality risk factors for substance misuse (Impulsivity, Sensation Seeking, Anxiety Sensitivity, and Hopelessness). While the SURPS has been used extensively with adolescents at risk for substance dependence, its properties with adult substance-dependent populations have been understudied. Further, the validity of the Bulgarian version of the SURPS has not been evaluated. The aims of the present study were to examine the factor structure of the Bulgarian version of the SURPS, its psychometric properties, and its ability to distinguish individuals with substance dependence from healthy controls.

**Methods:** Participants included 238 individuals ages 18 to 50 (45% female): 36 “pure” (i.e., mono-substance dependent) heroin users, 34 “pure” amphetamine users, 32 polysubstance users, 64 controls with no history of substance dependence, 43 unaffected siblings of heroin users, and 29 unaffected siblings of amphetamine users. We explored the factor structure of the Bulgarian version of the SURPS with confirmatory factor analyses, examined its reliability and validity, and tested for group differences between substance dependent and non-dependent groups.

**Results:** Confirmatory factor analyses (CFA) replicated the original four-factor model of the SURPS. The four subscales of the SURPS demonstrated good internal consistency (Cronbach's alphas ranged from 0.71 to 0.85) and adequate concurrent validity. Significant group differences were found on the Impulsivity and Sensation Seeking subscales, with the three substance dependent groups scoring higher than controls.

**Conclusions:** The SURPS is a valid instrument for measuring personality risk for substance use disorders in the Bulgarian population. The Bulgarian version of the SURPS demonstrates adequate to good reliability, concurrent validity, and predictive validity. Its ability to distinguish between groups with and without a history of substance dependence was specific to externalizing traits such as Impulsivity and Sensation Seeking, on which opiate, stimulant, and polysubstance dependent individuals scored higher than non-dependent controls.

## Introduction

Bulgaria is a key country for both drug production and trafficking, due to its strategic geographical position on the “Balkan Drug Route.” As a major European center for production of synthetic amphetamine-type stimulants and a key transit country for heroin trafficking, Bulgaria has a significant need for drug prevention, treatment, and research. The latest general population survey in the country, carried out in 2012, indicates that the average lifetime prevalence for illicit drug use was 10.3% for the general population aged 15–34 years (European Monitoring Centre for Drugs Drug Addiction, 2018), with cannabis being the most frequently used substance, followed by stimulants like ecstasy and amphetamines. Problem drug use in Bulgaria is related largely to opioid (primarily heroin) use. Intravenous injection is the primary method of heroin use and heroin and amphetamine use are the leading causes for treatment demand in outpatient and inpatient settings. Heroin users entering specialized drug treatment comprise 73% of all entrants and heroin remains the primary drug of choice for the majority of first-time treatment entrants (European Monitoring Centre for Drugs Drug Addiction, 2018). Licit substance use such as alcohol consumption per capita (in liters of pure alcohol for 2008–2010) is 17.9 for males and 5.3 for females, in comparison to 10.9 for the European region. Further, the 12-month prevalence of alcohol use disorders for males reached 12% in 2010 (WHO, 2014). Based on data from 2014, 35.4% of men and 19.9% of women in Bulgaria smoke tobacco, one of the highest rates in Europe (European Commission, 2017). Particularly alarming is the high level of substance use among adolescents in Bulgaria. According to the European School Survey Project on Alcohol and Other Drugs (ESPAD), the largest cross-national study on adolescent substance use in the world, Bulgarian 15- to 16-year old students report higher than average levels of use for six out of eight substance use variables (Kraus and Nociar, 2016). For cigarette use in the last 30 days, alcohol use in the last 30 days, and heavy episodic drinking in the last 30 days, the levels of use of Bulgarian adolescents exceed the ESPAD average by 10%. For lifetime cannabis use, Bulgarian students report one of the highest rates among the 35 ESPAD countries. Similarly, lifetime use of illicit drugs other than cannabis and lifetime use of new psychoactive substances (NPS) are almost twice as high for Bulgarian students relative to students in other European countries (Kraus and Nociar, 2016). Accordingly, not only is licit and illicit substance use and misuse high among Bulgarian adults, but trends among Bulgarian adolescents are also a public health concern.

From the multiple etiological risk factors for substance use disorders (SUDs), externalizing, and internalizing personality traits have been identified as some of the most salient risk factors. One of the most influential models in this field is the four-factor personality-based developmental model of SUD (Castellanos-Ryan and Conrod, 2012), which proposes that four distinct and independent lower-order (i.e., more narrowly defined) personality traits confer specific risk for different types of SUD and associated externalizing and internalizing psychopathology: *Impulsivity (IMP), Sensation Seeking (SS), Anxiety Sensitivity (AS), and Hopelessness (H)*. The four personality dimensions have been differentially associated with sensitivity to the positively and/or negatively reinforcing properties of different classes of drugs and in turn, susceptibility to different types of SUDs. The two externalizing traits (IMP & SS) have been associated with sensitivity to the positively-reinforcing incentive properties of drugs, with IMP associated specifically with stimulant misuse and SS with alcohol, cannabis, and polysubstance misuse (Conrod et al., 2000; Woicik et al., 2009; Castellanos-Ryan et al., 2011, 2014). In contrast, the two internalizing traits (AS & H) have been associated with preferential misuse of depressant and anxiolytic drugs, such as sedatives, opioids, and benzodiazepines, and a specific form of alcohol misuse related to coping mechanisms (Stewart et al., 1997; Woicik et al., 2009). AS has more recently also been associated with cannabis misuse (Keough et al., 2018).

These personality traits are measured with the 23-item Substance Use Risk Profile Scale (SURPS; Woicik et al., 2009). In contrast to personality measures like the NEO Five Factor Inventory (Costa and McCrae, 1992) or the Tridimensional Personality Questionnaire (Cloninger et al., 1991), which estimate a broad spectrum of general personality factors that are not specifically related to SUD vulnerability, the SURPS is specifically designed to assess lower-order (i.e., more narrowly defined) personality traits known to increase risk for SUD based on reinforcement-sensitivity models of substance use (Pihl and Peterson, 1995). It is comprised of items from a variety of other scales (e.g., Sensation Seeking Scale, Anxiety Sensitivity Index, Beck Hopelessness Scale, Eysenck Impulsiveness, and Venturesomeness Scales, etc.) that have shown the highest predictive validity for substance misuse among adolescents. Though the four personality traits assessed with the SURPS could be assessed independently with the different scales, the SURPS has the advantage that it incorporates the most predictive items of these scales into a single measurement tool. The four SURPS personality risk factors are successfully targeted by selective personality targeted brief interventions (Conrod et al., 2010), shown to delay onset and reduce alcohol and drug use among adolescents.

The SURPS has been used with different populations, including adolescents (Chandrika Ismail et al., 2009; Woicik et al., 2009; Malmberg et al., 2010; Siu, 2010; Krank et al., 2011; Castonguay-Jolin et al., 2013; Castellanos-Ryan et al., 2014; Memetovic et al., 2014; Robles-Garcia et al., 2014; Jurk et al., 2015; Ali et al., 2016; Newton et al., 2016), undergraduate and graduate college students (Omiya et al., 2015), and non-substance using adults (Canfield et al., 2015). However, its properties with adult substance-dependent populations have been understudied. To our knowledge, only two studies to date have used the SURPS with substance dependent individuals. In the first of these studies, Schlauch et al. (2015) demonstrated good psychometric properties of the SURPS among inpatient substance users. The second study was focused on the predictive validity of the scale with incarcerated male offenders and found that sensation seeking and anxiety sensitivity were associated with institutional drug use (Hopley and Brunelle, 2016). Notably, no study to date has examined the properties of the SURPS among community samples of substance dependent individuals.

To address this gap in the literature and to evaluate the psychometric properties of the Bulgarian version of the scale, we administered the SURPS to a Bulgarian community sample of substance dependent individuals and non-dependent controls. The main goals of the present study were to examine the factor structure of the Bulgarian version of the SURPS, evaluate its reliability and validity, and assess its ability to distinguish individuals with substance dependence from non-dependent controls. Based on previous applications of the SURPS to predominately at-risk adolescent and college student populations, we hypothesized that we will similarly identify four factors in this new sample of Bulgarian substance dependent individuals. We also predicted that the four SURPS subscales will correlate with similar vulnerability scales and predict SUDs and related outcomes.

## Materials and methods

### Participants

Participants were recruited from a larger ongoing study on neurocognitive functioning among substance dependent individuals in Bulgaria, via flyers placed at substance abuse clinics, nightclubs, bars, and cafes, as well as, through the study's web page and Facebook page. Participants were initially screened via telephone or in-person on their medical and substance use histories.

Participants had to meet the following inclusion criteria: (1) age between 18 and 50 years; (2) Raven's Progressive Matrices (Raven, 2000) estimated IQ > 75; (3) minimum of 8th grade education; (4) no history of central nervous system illness or injury; (5) HIV-seronegative status, in order to control for the potential confounding effects of HIV on neurocognitive function; (6) negative breathalyzer test for alcohol and negative urine toxicology screen for amphetamines, methamphetamines, cocaine, opiates, methadone, cannabis, benzodiazepines, barbiturates, and MDMA. Exclusion criteria included history of neurologic illness or injury, open head injury of any type, closed head injury with loss of consciousness >30 min, presence of psychotic or mood disorders, and current use of antipsychotic medication. All participants were HIV seronegative (determined by rapid HIV testing) and no participants were on opioid substitution therapy.

We screened 508 individuals (63.2% male) via telephone on their medical and substance use histories. Of those, 238 individuals participated and 270 individuals did not participate in the study. The main reasons for non-participation were: (1) unwillingness to abstain from drug use prior to the study visits (*n* = 64; 23.7%); (2) control participants in excess of our target sample size for controls (*n* = 45; 16.7%); (3) being currently on methadone maintenance treatment (*n* = 41; 15.2%); (4) substance dependent participants who do not have any siblings (*n* = 39; 14.4%); (5) not interested in participating (*n* = 35; 13%); (6) the participant does not meet full criteria for SUD (*n* = 26; 9.6%); (7) history of neurologic illness or injury (*n* = 9; 3.4%); (8) ethnicity other than Bulgarian (*n* = 6; 2.2%), in order to increase the sample homogeneity for the genetic component of the larger study; and (9) presence of psychotic or mood disorders (*n* = 5; 1.9%). There were no significant differences in key variables such as age and sex between individuals who participated in the study and those who were only screened.

The final sample included 238 individuals (55% male), of whom 36 met DSM-IV (American Psychiatric Association, 2000) criteria for lifetime mono-dependence on heroin, 34 met criteria for lifetime mono-dependence on amphetamines, and 32 met lifetime criteria for dependence on more than one substance, including heroin and/or amphetamines. The sample also included 64 control individuals with no history of substance abuse or dependence, as well as, 43 unaffected siblings of heroin users and 29 unaffected siblings of amphetamine users who were treated as controls. At the time of testing, the majority of substance dependent participants were in protracted abstinence (i.e., sustained full remission by DSM-IV criteria for 12 months or longer). All participants were ethnic Bulgarians. Please see Table 1 for participant characteristics by group.

**Table 1:** Participant characteristics by group (*N* = 238).

	**Controls** **(*n* = 64)**	**Heroin** **(*n* = 36)**	**Amphetamine** **(*n* = 34)**	**Polysubstance** **(*n* = 32)**	**Heroin Siblings** **(*n* = 43)**	**Amphetamine Siblings** **(*n* = 29)**
Age, mean (SD)	28.55 (7.62)	35.31 (5.31)	27.47 (5.89)	30.34 (6.67)	33.28 (7.76)	27.72 (8.77)
**SEX**, ***N*****(%)**						
Male	26 (40.6)	26 (72.2)	22 (64.7)	25 (78.1)	21 (48.8)	12 (41.4)
Female	38 (59.4)	10 (27.8)	12 (35.3)	7 (21.9)	22 (51.2)	17 (58.6)
Estimated IQ, mean (SD)	111.92 (12.68)	105.41 (11.84)	107.97 (12.88)	109.56 (11.78)	111.07 (12.61)	112.00 (14.35)
Years of education, mean (SD)	15.73 (2.26)	13.00 (2.65)	13.58 (2.45)	13.25 (3.13)	15.12 (2.77)	14.64 (2.25)
Addiction Severity Index Psychiatric Composite, mean (SD)	0.07 (0.14)	0.06 (0.12)	0.13 (0.17)	0.14 (0.17)	0.06 (0.14)	0.10 (0.17)
**NUMBER OF DSM-IV ABUSE SYMPTOMS, MEAN (*****SD*****)**						
Alcohol	0.03 (0.25)	0.36 (0.64)	0.85 (1.05)	1.19 (1.33)	0.00 (0.00)	0.03 (0.19)
Cannabis	0.03 (0.25)	0.81 (0.98)	1.44 (1.21)	1.87 (1.04)	0.14 (0.47)	0.24 (0.76)
Heroin	0.00 (0.00)	2.89 (1.09)	0.00 (0.00)	1.88 (1.72)	0.00 (0.00)	0.00 (0.00)
Amphetamine	0.00 (0.00)	0.17 (0.51)	1.68 (1.09)	2.25 (1.30)	0.00 (0.00)	0.00 (0.00)
**NUMBER OF DSM-IV DEPENDENCE SYMPTOMS, MEAN (*****SD*****)**						
Alcohol	0.05 (0.28)	0.47 (0.77)	0.71 (0.87)	1.50 (1.90)	0.12 (0.32)	0.07 (0.26)
Cannabis	0.09 (0.39)	0.67 (0.86)	1.21 (0.81)	2.69 (1.79)	0.09 (0.29)	0.28 (0.88)
Heroin	0.00 (0.00)	5.86 (1.07)	0.00 (0.00)	3.56 (3.03)	0.00 (0.00)	0.00 (0.00)
Amphetamine	0.00 (0.00)	0.22 (0.64)	4.24 (1.72)	4.38 (2.35)	0.00 (0.00)	0.00 (0.00)
**YEARS OF USE, MEAN (*****SD*****)**						
Heroin	0.00 (0.00)	6.43 (2.74)	0.00 (0.00)	4.80 (5.08)	0.00 (0.00)	0.06 (0.32)
Amphetamine	0.02 (0.19)	0.64 (2.66)	4.61 (3.13)	4.12 (3.83)	0.00 (0.00)	0.00 (0.00)
**YEARS SINCE LAST MEETING DEPENDENCE, MEAN (*****SD*****)**						
Heroin	–	9.87 (6.29)	–	5.74 (5.46)	–	–
Amphetamine	–	13.00 (0.00)^*^	4.19 (3.51)	4.88 (4.80)	–	–

Participants who do not meet dependence criteria were not asked the “years since last meeting dependence criteria” question and these cells are accordingly empty.

* *One individual in the heroin group met criteria for amphetamine dependence 13 years ago*.

### Procedures

The study was approved by the Institutional Review Boards of Virginia Commonwealth University and the Medical University-Sofia on behalf of the Bulgarian Addictions Institute. All subjects gave written informed consent in accordance with the Declaration of Helsinki. Participants who met the inclusion criteria were contacted via telephone and invited to participate in the study. Briefly, after signing an informed consent form participants underwent urine drug screens and a Breathalyzer test for alcohol. Then they completed two study sessions of ~4 h each, conducted on two separate days, which included clinical interviews, self-report scales, and a battery of computerized neurocognitive measures. The first session included assessment of substance use disorders, externalizing psychopathology (e.g., psychopathy, ASPD, ADHD) and intelligence. The second session included neurocognitive tasks of impulsivity and decision-making and self-report measurements of personality (e.g., aggression) and internalizing psychopathology (e.g., anxiety, alexithymia).

### Instruments

Some of the self-report instruments (e.g., Beck Depression Inventory, State Trait Anxiety Inventory, Sensation Seeking Scale) were already translated and in use in Bulgaria. The rest of the measures were translated into Bulgarian by the senior author (JV), a clinical neuropsychologist and a native Bulgarian speaker. The measures were then back-translated into English by Bulgarian psychiatrists and psychologists, including co-authors GV and KB. The translations were reviewed by one of the authors and by two independent mental health professionals/psychologists and psychiatrists to attain consensus for language adaptation for each item.

#### Substance use risk profile scale

The *Substance Use Risk Profile Scale (SURPS;* Woicik et al., 2009) is a 23-item self-report scale assessing 4 personality traits associated with increased risk for substance misuse. The scale consists of 4 subscales: Impulsivity (5 items), Sensation seeking (6 items), Hopelessness (7 items), and Anxiety sensitivity (5 items). The instrument was administered in paper-and-pencil format. Respondents were asked to mark the level to which they agreed with each item on a 4-point Likert Scale ranging from 1 (strongly disagree) to 4 (strongly agree). All but one of the items in the Hopelessness subscale were reverse scored.

#### Addiction severity index—lite version

The *Addiction Severity Index—Lite Version (ASI-Lite;*McLellan et al., 1992) is a semi-structured clinical interview intended to assesses addiction severity and level of functioning across 7 domains: medical, employment, alcohol use, drug use, legal, family/social, and psychiatric. Because we wanted to control for global psychiatric functioning, we used the composite score from the psychiatric section only, which contains questions determining whether participants have had past or present significant problems with depression, anxiety, violent behavior, and suicidal thoughts/behavior, as well as, whether they are currently taking or have ever taken any psychiatric medication. The composite score ranges from 0 to 1, with higher score indicating greater problem severity.

#### Barratt impulsiveness scale

The *Barratt Impulsiveness Scale (BIS-11*; Patton et al., 1995) is a 30-item self-report instrument that measures impulsive personality traits in three dimensions: attentional, motor, and non-planning impulsiveness. Respondents indicate the extent to which they agree with each item, ranging from 1 (rarely/never) to 4 (almost always/always). In the present sample the total scale yielded good internal consistency (α = 0.84).

#### UPPS-P impulsive behavior scale

The *UPPS-P Impulsive Behavior Scale* (Lynam et al., 2006) is a 59-item questionnaire that assesses five personality dimensions of impulsive behavior: (lack of) premeditation, (lack of) perseverance, sensation seeking, negative urgency, and positive urgency (Cyders and Smith, 2007), with items rated on a 4-point scale from strongly agree to strongly disagree. The UPPS-P in the current sample showed excellent internal consistency (α = 0.94).

#### Sensation seeking scale-V

The *Sensation Seeking Scale-V (SSS-V;* Zuckerman, 1996) is a 40-item forced choice measure comprised of four subscales related to sensation-seeking behaviors: thrill and adventure seeking, experience seeking, disinhibition, and boredom susceptibility. We used the existing (unpublished) Bulgarian version of the scale. In the current sample the total scale exhibited good internal consistency (α = 0.83).

#### Beck depression inventory-II

The *Beck Depression Inventory-II (BDI-II*; Beck et al., 1996) is a 21-item scale that measures severity of depression symptoms during the last 2 weeks and asks participants to rate the extent to which they endorse each symptom on a 4-point Likert Scale. We used the existing (unpublished) Bulgarian version of the scale. In the current sample the BDI-II showed good internal consistency (α = 0.87).

#### Anxiety sensitivity index

The *Anxiety Sensitivity Index (ANXSI;* Reiss et al., 1986) is a 16-item, 5-point Likert scale that measures anxiety sensitivity as a global construct composed of several factors differentiating fear of specific anxiety symptoms and associated catastrophic consequences (Olthuis et al., 2014). The scale had good internal consistency in the current sample (α = 0.85).

#### State-trait anxiety inventory

The *State-Trait Anxiety Inventory (STAI;* Spielberger et al., 1983) is a 4-point Likert scale that consists of 40 items. The STAI-Trait scale had 20 statements that ask how participants feel in general, whereas the STAI-State scale had 20 statements that ask how they feel at the moment. We used the existing Bulgarian adaption of the scale (Shtetinski and Paspalanov, 2008). Both the state and the trait sections of the scale had excellent internal consistency in this sample (α = 0.9 and α = 0.91).

#### Psychopathy checklist: screening version

The *Psychopathy Checklist: Screening Version (PCL:SV;* Hart et al., 1995) is a 12-item, interviewer-completed scale based on a semi-structured interview. The Bulgarian version of the instrument (Wilson et al., 2014) was used to assess psychopathy, indexed with an interpersonal/affective factor (F1) and an antisocial/lifestyle factor (F2) based on the original two-factor model of psychopathy (Hare, 1991). Interviews and psychopathy ratings were conducted by an experienced team of research assistants and psychologists who were initially trained for reliability and supervised closely by JV, the author of the Bulgarian version of the Psychopathy Checklist-Revised (PCL-R) with its publisher Multi Health Systems. Two members of the research team (GV and KB) were further trained directly by Robert Hare, the author of the PCL in a training workshop. In line with our earlier findings (Wilson et al., 2014), the PCL:SV showed good internal consistency for the total scale (α = 0.92) and its two subscales (α = 0.84 and α = 0.89) in the current sample.

#### Wender utah rating scale

The *Wender Utah Rating Scale* (*WURS;* Ward et al., 1993) is a self-report scale used to evaluate adults for childhood symptoms of ADHD. Respondents were asked to retrospectively evaluate the presence and severity of childhood symptoms of ADHD. We used the recently validated 25-item Bulgarian version of the scale (Nedelchev et al., 2016). The scale had excellent internal consistency (α = 0.93) in the current sample.

#### Toronto alexithymia scale

The *Toronto Alexithymia Scale (TAS-20;* Bagby et al., 1994) is a 20-item scale designed to measure alexithymia associated with difficulties identifying and describing one's own feelings (Leising et al., 2009). The scale consists of three factors: Factor 1, comprised of items assessing the capacity to identify feelings and to distinguish between feelings and bodily sensations of emotional arousal; Factor 2, comprised of items reflecting the inability to communicate feelings to other people; and Factor 3, comprised of items assessing externally oriented thinking. We used the recently translated and validated Bulgarian version of the TAS-20 (Popov et al., 2016). The scale had good internal consistency in the present sample (α = 0.82).

#### Fagerstrom test for nicotine dependence

The *Fagerstrom Test for Nicotine Dependence (FTND;* Heatherton et al., 1991) was administered to assess nicotine dependence or the intensity of physical addiction to nicotine (Fagerstrom, 1978).

#### Structured clinical interview for DSM-IV

Finally, the Substance Abuse and the Antisocial Personality Modules of the *Structured Clinical Interview for DSM-IV (SCID-I;* First and Gibbon, 2004) were used to obtain participants' history of substance abuse and dependence, the total number of substance dependent diagnoses, history of conduct disorder (CD), and history of antisocial personality disorder (ASPD). We also tabulated years of heroin and amphetamine use and length of abstinence, indexed as number of days (converted to years) since last met DSM-IV heroin or amphetamine dependence criteria.

### Statistical analyses

We examined the factor structure, reliability, and validity of the SURPS. The factor structure was assessed by confirmatory factor analysis (CFA) in the OpenMx software package (version 2.7.10; Boker et al., 2011, 2016; Neale et al., 2015) using R Version 3.4.1 (R Development Core Team, 2013). We relied on a variety of relative and absolute goodness-of-fit indices to examine the fit of the CFA: the Non-Normed Fit Index (NNFI, also known as TLI), the Comparative Fit Index (CFI), and the Root Mean Square of Approximation (RMSEA) (Schreiber et al., 2006). For the TLI and CFI, a value above 0.90 is considered an indication of acceptable fit. For the RMSEA, a value < 0.05 indicates acceptable fit. We note however that the absolute fit index (RMSEA) may be more reliable for our sample size (>200) than the relative fit indices (TLI and CFI) (Fan et al., 1999).

Internal consistency was assessed using Cronbach's alpha. Convergent and discriminant validity was assessed by Pearson correlations between the SURPS subscales and theoretically related measures (BIS, SSS, UPPS-P, BDI-II, ANXSI, STAI-S, and STAI-T). Because we included siblings, the nested structure of the data was accounted for by using the *statsBy* function in the *psych* package (Revelle, 2018). This simple function provides basic descriptive statistics for two level models, where we were simultaneously able to control for the sibling data and sex. The observed correlations are decomposed into the within group and between group correlations.

Predictive validity was assessed with generalized estimating equations (GEE) generalized linear models (Liang and Zeger, 1986) via the *geeglm* function in the *geepack* package (Højsgaard et al., 2016) in R to determine whether the SURPS subscales predict substance use disorders (SUDs) and related outcomes [PCL: SV; WURS; TAS-20; BDI-II; FTND; number of SCID symptoms for alcohol abuse and dependence, cannabis abuse and dependence, heroin abuse and dependence, amphetamine abuse and dependence; total number of DSM-IV substance dependence diagnoses; number of SCID symptoms for conduct disorder and antisocial personality disorder; number of days of heroin and amphetamine use (converted to years); and number of days since last meeting DSM-IV dependence criteria (converted to years)]. GEE models are an extension of generalized linear models to the analysis of longitudinal data or data that otherwise violates the assumption of independence (Liang and Zeger, 1986). In these models, the assumption of normal distributions is also relaxed. We included age, sex, and global psychiatric status (assessed by the ASI Psychiatric Composite) as covariates in all models.

Finally, the ability of the SURPS subscales to distinguish among the four groups (control, heroin, amphetamine, and polysubstance users) was determined using the *anova* function (Fox, 2018) in the *car* package (Fox and Weisberg, 2018) by providing the function with fitted *geeglm* models. For generalized linear models such as these, the *anova* function calculates the Wald chi-square. Tukey's *post-hoc* comparisons were then conducted using the *pairs* procedure in the *emmeans* package (Emmeans Package, 2018).

## Results

### Factor structure

**Figure 1:**
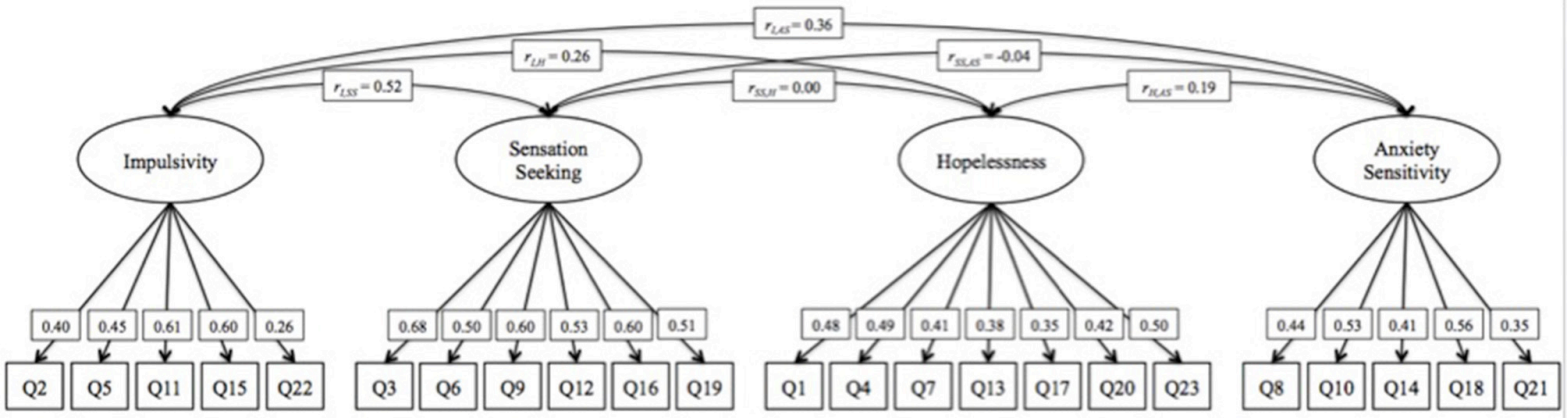
4-factor CFA model with correlated factors.

We tested a 4-factor CFA to assess the factor structure of the SURPS, allowing for correlated factors (Figure 1). This model demonstrated acceptable model fit (RMSEA = 0.038; CFI = 0.88; TLI = 0.86). Although the CFI and TLI fit indices were slightly lower than the recommended value of 0.9, relative fit indices may not be reliable indicators with sample sizes > 200. In such cases, the absolute fit index (RMSEA) is generally more reliable and we therefore relied more heavily on this index than on the others (Fan et al., 1999). Factor loadings ranged between 0.26 and 0.61 for impulsivity; 0.50–0.68 for sensation seeking; 0.35–0.50 for hopelessness; and 0.35–0.56 for anxiety sensitivity. Inspection of the factor correlations showed that the IMP factor correlated highly with SS (*r* = 0.52) and moderately with H (*r* = 0.26) and AS (*r* = 0.36); H and AS were moderately correlated (*r* = 0.19); and the correlations between SS and H (*r* = 0.00) and between SS and AS (*r* = −0.04) were negligible.

### Reliability and validity

#### Internal consistency

The alpha coefficients for each subscale were as follows: IMP: 0.71 (95% CI: 0.68–0.75); SS: 0.78 (95% CI: 0.75–0.80); H: 0.85 (95% CI: 0.83–0.87); AS: 0.73 (95% CI: 0.70–0.77). All coefficients were above the recommended value of 0.70, demonstrating adequate reliability (Cronbach, 1951; Nunnally et al., 1967).

#### Convergent and discriminant validity

The Pearson correlation matrix between each SURPS subscale and other measures purported to assess the same construct are shown in Table 2. The SURPS-IMP correlated highly with the BIS-11 and UPPS-P (*r* = 0.69 and 0.71, respectively); the SURPS-SS correlated highly with the SSS-V and UPPS-P (*r* = 0.53 and 0.70, respectively); the SURPS-H correlated moderately with the BDI-II (*r* = 0.46); and the SURPS-AS correlated modestly to moderately with the ANXSI (*r* = 0.52), STAI-S (*r* = 0.23), and STAI-T (*r* = 0.36). Although the correlation between the SURPS-AS and the STAI-S (*r* = 0.23) was lower than other correlations, it is still significant and more highly correlated with the ANXSI. Taken together, these results demonstrate good convergent validity.

**Table 2:** Convergent and discriminant validity: Pearson correlations.

	**1**.	**2**.	**3**.	**4**.	**5**.	**6**.	**7**.	**8**.	**9**.	**10**.	**11**.	**12**.	**13**.
1. Age	1.00											
2. ASI Psychiatric Composite	0.01	1.00										
3. SURPS Impulsivity	0.03	**0.25**	1.00									
4. SURPS Sensation Seeking	−0.03	0.13	**0.36**	1.00								
5. SURPS Hopelessness	**0.15**	**0.24**	**0.26**	0.00	1.00							
6. SURPS Anxiety Sensitivity	0.12	**0.16**	**0.34**	−0.04	**0.18**	1.00						
7. Barratt Impulsiveness Scale-11	–**0.07**	**0.25**	**0.69**	**0.36**	**0.32**	**0.21**	1.00					
8. UPPS-P Impulsive Behavior Scale	0.02	**0.25**	**0.71**	**0.53**	**0.29**	**0.20**	**0.66**	1.00				
9. Sensation Seeking Scale	–**0.20**	0.02	**0.31**	**0.70**	0.06	–**0.15**	**0.39**	**0.43**	1.00			
10. Beck Depression Inventory-II	0.00	**0.39**	**0.35**	**0.15**	**0.46**	**0.33**	**0.40**	**0.34**	**0.09**	1.00		
11. Anxiety Sensitivity Index	0.06	**0.28**	**0.28**	0.00	**0.09**	**0.52**	**0.15**	**0.19**	−0.04	**0.34**	1.00	
12. State Anxiety (STAI-S)	0.00	**0.31**	**0.31**	0.06	**0.52**	**0.23**	**0.32**	**0.28**	**0.10**	**0.52**	**0.24**	1.00
13. Trait Anxiety (STAI-T)	−0.04	**0.44**	**0.39**	**0.12**	**0.61**	**0.36**	**0.42**	**0.37**	**0.11**	**0.70**	**0.39**	**0.63**	1.00

Bolded correlations are significant at p < 0.05. Correlations were adjusted for sibling data and gender. ASI, Addiction Severity Index.

Regarding discriminant validity, the SURPS-IMP was significantly correlated with all of the other measures, but was correlated with the internalizing measures (BDI-II, ANXSI, STAI-S, and STAI-T) to a much lesser degree (*r* = 0.28–0.39) than with the impulsivity measures. The SURP-SS was very modestly correlated with the BDI-II (*r* = 0.15) and STAI-T (*r* = 0.12). The SURPS-H was modestly to moderately correlated with the impulsivity measures (*r* = 0.06–0.32), but was correlated with other internalizing measures to a greater degree, such as the STAI-S (*r* = 0.52) and STAI-T (*r* = 0.61). Finally, the SURPS-AS demonstrated perhaps the greatest amount of discriminant validity, as it was significantly negatively correlated with the SSS (*r* = −0.15) and modestly correlated with the other impulsivity measures. Overall, the magnitude of the correlations were highest between the SURPS subscales and measures purported to measure similar constructs (i.e., SURPS-IMP and SURPS-SS with other externalizing measures; SURPS-H and SURPS-AS with other internalizing measures) and were considerably lower for measures purported to measure different constructs, demonstrating good discriminant validity.

#### Predictive validity

The results of the GEE generalized linear models are shown in Table 3. The majority of the theoretically related measures were significantly associated with their specific subscales as expected. For example, the SURPS-H was significantly associated with depression, measured with the BDI-II, and the SURPS-IMP was significantly associated with the WURS, indexing childhood symptoms of ADHD. Additionally, the SURPS-IMP and SURPS-SS were consistently associated with a number of clinical measures of SUD as assessed by the SCID-IV. The SURPS-AS was also significantly associated with nicotine dependence, number of symptoms of cannabis abuse, amphetamine dependence, antisocial personality disorder, and years of heroin use. However, somewhat surprisingly, none of the SURPS subscales were significantly associated with heroin abuse, heroin dependence, and years since last met DSM-IV criteria for heroin and amphetamine dependence.

**Table 3:** Predictive validity: Generalized estimating equation (GEE) generalized linear model results.

	**Estimate (SE)**	**Wald**	**p**
**OUTCOME** = **PSYCHOPATHY CHECKLIST (N** = **232)**			
SURPS-Impulsivity	**0.87 (0.16)**	**30.64**	<**0.001**
SURPS-Sensation Seeking	−0.01 (0.11)	0.02	0.896
SURPS-Hopelessness	–**0.54 (0.12)**	**19.35**	<**0.001**
SURPS-Anxiety Sensitivity	0.20 (0.12)	2.63	0.105
**OUTCOME** = **INTERPERSONAL/AFFECTIVE (FACTOR 1) OF**			
**THE PSYCHOPATHY CHECKLIST (N** = **232)**			
SURPS-Impulsivity	−1.29 (2.54)	0.26	0.613
SURPS-Sensation Seeking	1.82 (1.09)	2.79	0.095
SURPS-Hopelessness	0.99 (0.92)	1.15	0.283
SURPS-Anxiety Sensitivity	**1.71 (0.77)**	**4.94**	**0.026**
**OUTCOME** = **IMPULSIVE/ANTISOCIAL (FACTOR 2) OF THE**			
**PSYCHOPATHY CHECKLIST (N** = **231)**			
SURPS-Impulsivity	**0.49 (0.10)**	**25.28**	<**0.001**
SURPS-Sensation Seeking	0.07 (0.06)	1.35	0.246
SURPS-Hopelessness	–**0.16 (0.07)**	**6.07**	**0.014**
SURPS-Anxiety Sensitivity	0.12 (0.08)	2.23	0.135
**OUTCOME** = **WENDER UTAH RATING SCALE FOR ADHD (N** = **238)**			
SURPS-Impulsivity	**2.47 (0.46)**	**29.21**	<**0.001**
SURPS-Sensation Seeking	−0.04 (0.30)	0.02	0.884
SURPS-Hopelessness	−0.07 (0.31)	0.06	0.810
SURPS-Anxiety Sensitivity	0.55 (0.51)	1.20	0.273
**OUTCOME** = **TORONTO ALEXITHYMIA SCALE (N** = **238)**			
SURPS-Impulsivity	**0.79 (0.24)**	**10.52**	**0.001**
SURPS-Sensation Seeking	0.18 (0.14)	1.75	0.186
SURPS-Hopelessness	**1.13 (0.18)**	**41.94**	<**0.001**
SURPS-Anxiety Sensitivity	**0.67 (0.21)**	**10.33**	**0.001**
**OUTCOME** = **BECK DEPRESSION INVENTORY-II (N** = **237)**			
SURPS-Impulsivity	0.25 (0.14)	2.99	0.083
SURPS-Sensation Seeking	0.02 (0.09)	0.03	0.869
SURPS-Hopelessness	**0.67 (0.12)**	**29.21**	<**0.001**
SURPS-Anxiety Sensitivity	0.00 (0.13)	0.00	0.994
**OUTCOME** = **FAGERSTROM TEST OF NICOTINE DEPENDENCE (N** = **238)**			
SURPS-Impulsivity	0.15 (0.09)	3.00	0.083
SURPS-Sensation Seeking	0.02 (0.05)	0.21	0.646
SURPS-Hopelessness	0.01 (0.06)	0.05	0.818
SURPS-Anxiety Sensitivity	**0.15 (0.07)**	**4.61**	**0.032**
**OUTCOME** = **SCID # SX. ALCOHOL ABUSE (N** = **238)**			
SURPS-Impulsivity	**0.04 (0.02)**	**4.02**	**0.045**
SURPS-Sensation Seeking	**0.03 (0.01)**	**3.96**	**0.047**
SURPS-Hopelessness	−0.01 (0.02)	0.15	0.694
SURPS-Anxiety Sensitivity	0.01 (0.02)	0.14	0.704
**OUTCOME** = **SCID # SX. ALCOHOL DEPENDENCE (N** = **238)**			
SURPS-Impulsivity	**0.04 (0.02)**	**4.43**	**0.035**
SURPS-Sensation Seeking	0.03 (0.02)	3.74	0.053
SURPS-Hopelessness	−0.02 (0.02)	0.59	0.441
SURPS-Anxiety Sensitivity	0.04(0.03)	2.67	0.102
**OUTCOME** = **SCID # SX. CANNABIS ABUSE (N** = **238)**			
SURPS-Impulsivity	**0.05 (0.03)**	**4.05**	**0.044**
SURPS-Sensation Seeking	**0.05 (0.02)**	**6.65**	**0.010**
SURPS-Hopelessness	–**0.04 (0.02)**	**5.32**	**0.021**
SURPS-Anxiety Sensitivity	**0.05 (0.02)**	**5.25**	**0.022**
**OUTCOME** = **SCID # SX. CANNABIS DEPENDENCE (N** = **238)**			
SURPS-Impulsivity	**0.07 (0.03)**	**5.01**	**0.025**
SURPS-Sensation Seeking	**0.06 (0.02)**	**8.15**	**0.004**
SURPS-Hopelessness	−0.03 (0.02)	1.52	0.218
SURPS-Anxiety Sensitivity	0.05 (0.03)	3.47	0.063
**OUTCOME** = **SCID # SX. HEROIN ABUSE (N** = **238)**			
SURPS-Impulsivity	0.04 (0.03)	1.43	0.232
SURPS-Sensation Seeking	0.04 (0.02)	2.67	0.102
SURPS-Hopelessness	0.01 (0.02)	0.14	0.712
SURPS-Anxiety Sensitivity	0.06 (0.03)	3.00	0.083
**OUTCOME** = **SCID # SX. HEROIN DEPENDENCE (N** = **238)**			
SURPS-Impulsivity	0.11 (0.06)	3.33	0.068
SURPS-Sensation Seeking	0.05 (0.04)	1.61	0.204
SURPS-Hopelessness	−0.01 (0.04)	0.12	0.730
SURPS-Anxiety Sensitivity	0.07 (0.06)	1.49	0.223
**OUTCOME** = **SCID # SX. AMPHETAMINE ABUSE (N** = **238)**			
SURPS-Impulsivity	**0.49 (0.14)**	**12.91**	<**0.001**
SURPS-Sensation Seeking	−0.01 (0.08)	0.01	0.912
SURPS-Hopelessness	–**0.70 (0.12)**	**32.27**	<**0.001**
SURPS-Anxiety Sensitivity	−0.10 (0.12)	0.75	0.387
**OUTCOME** = **SCID # SX. AMPHETAMINE DEPENDENCE (N** = **238)**			
SURPS-Impulsivity	**0.14 (0.06)**	**6.32**	**0.012**
SURPS-Sensation Seeking	0.06 (0.04)	2.00	0.157
SURPS-Hopelessness	−0.05 (0.04)	1.31	0.252
SURPS-Anxiety Sensitivity	**0.11 (0.05)**	**5.47**	**0.019**
**OUTCOME** = **TOTAL # OF SCID DEPENDENCE DIAGNOSES (N** = **238)**			
SURPS-Impulsivity	**0.10 (0.03)**	**9.99**	**0.002**
SURPS-Sensation Seeking	0.02 (0.02)	0.77	0.381
SURPS-Hopelessness	−0.03 (0.02)	1.36	0.244
SURPS-Anxiety Sensitivity	0.04 (0.03)	2.12	0.145
**OUTCOME** = **SCID # SX. OF CONDUCT DISORDER (N** = **238)**			
SURPS-Impulsivity	**0.17 (0.05)**	**10.82**	**0.001**
SURPS-Sensation Seeking	0.02 (0.03)	0.51	0.477
SURPS-Hopelessness	–**0.14 (0.04)**	**12.29**	<**0.001**
SURPS-Anxiety Sensitivity	0.08 (0.04)	3.34	0.067
**OUTCOME** = **SCID # SX. OF ANTISOCIAL PERSONALITY DISORDER**			
(**N** = **238**)			
SURPS-Impulsivity	**0.24 (0.05)**	**24.04**	<**0.001**
SURPS-Sensation Seeking	0.01 (0.03)	0.18	0.674
SURPS-Hopelessness	–**0.14 (0.03)**	**19.55**	<**0.001**
SURPS-Anxiety Sensitivity	**0.10 (0.04)**	**6.31**	**0.012**
**OUTCOME** = **YEARS OF HEROIN USE (N** = **234)**			
SURPS-Impulsivity	**0.19 (0.08)**	**5.65**	**0.017**
SURPS-Sensation Seeking	0.07 (0.05)	1.52	0.218
SURPS-Hopelessness	0.01 (0.07)	0.04	0.832
SURPS-Anxiety Sensitivity	**0.17 (0.08)**	**4.72**	**0.030**
**OUTCOME** = **YEARS OF AMPHETAMINE USE (N** = **235)**			
SURPS-Impulsivity	0.13 (0.07)	3.77	0.052
SURPS-Sensation Seeking	0.09 (0.05)	3.49	0.062
SURPS-Hopelessness	−0.08 (0.06)	1.82	0.178
SURPS-Anxiety Sensitivity	0.12 (0.07)	2.70	0.100
**OUTCOME** = **LENGTH OF ABSTINENCE, HEROIN DEPENDENCE (N** = 56)^*^			
SURPS-Impulsivity	0.01 (0.25)	–	0.959
SURPS-Sensation Seeking	−0.15 (0.18)	–	0.395
SURPS-Hopelessness	−0.30 (0.19)	–	0.108
SURPS-Anxiety Sensitivity	−0.06 (0.27)	–	0.820
**OUTCOME** = **LENGTH OF ABSTINENCE, AMPHETAMINE DEPENDENCE**			
**(N** = 58)^*^			
SURPS-Impulsivity	0.17 (0.21)	–	0.410
SURPS-Sensation Seeking	0.05 (0.16)	–	0.768
SURPS-Hopelessness	0.23 (0.15)	–	0.124
SURPS-Anxiety Sensitivity	0.19 (0.22)	–	0.377

* *The length of abstinence models included only those in the respective drug groups and polysubstance group; because no siblings were included, regular linear regressions were run for those models. All models were adjusted for age, sex, and global psychiatric status (ASI Psychiatric Composite). SCID, Structured Clinical Interview for DSM-IV; Sx., symptoms. Bolded values indicate significance at p < 0.05*.

#### Comparison of SURPS scores across SUD groups and controls

The results of the ANOVAs and Tukey's *post-hoc* comparisons are shown in Table 4, along with the N for each group, the group means, and standard errors. The overall ANOVAs were significant for the SURPS-IMP (*p* < 0.001), SURPS-SS (*p* < 0.001), and the SURPS-AS (*p* = 0.003), but not for the SURPS-H (*p* = 0.510). Tukey's *post-hoc* comparisons showed that the SURPS-IMP and SURPS-SS were able to distinguish between groups, with each of the three SUD groups scoring higher than controls. The SURPS-H and SURPS-AS did not show any significant group differences.

**Table 4:** Comparison of SURPS scores across substance dependent groups and controls.

**SURPS Subscale**	**Group**	**N**	**Mean**	**SE**	**Wald *X^2^***	***p***	***post-hocs***
Impulsivity	Control (0)	136	9.53	0.21	36.9	< 0.001	1, 2, 3 > 0
	Heroin (1)	36	11.22	0.43		
	Amphetamine (2)	34	11.06	0.44		
	Polysubstance (3)	32	12.09	0.43		
Sensation Seeking	Control (0)	136	14.89	0.33	20.6	< 0.001	1, 2, 3 > 0
	Heroin (1)	36	16.75	0.58		
	Amphetamine (2)	34	17.09	0.59		
	Polysubstance (3)	32	17.44	0.66		
Hopelessness	Control (0)	136	12.58	0.27	2.3	0.510	N/A
	Heroin (1)	36	13.03	0.46		
	Amphetamine (2)	34	12.24	0.58		
	Polysubstance (3)	32	13.38	0.73		
Anxiety Sensitivity	Control (0)	136	10.99	0.23	13.8	0.003	N/A
	Heroin (1)	36	12.00	0.36		
	Amphetamine (2)	34	12.21	0.49		
	Polysubstance (3)	32	12.22	0.44		

*Only post-hoc comparisons that are significant (p ≤ 0.05) after adjusting p-values for multiple testing are presented. SE, standard error*.

## Discussion

The aims of the present study were to examine the factor structure and psychometric properties of the Bulgarian version of the SURPS and its ability to distinguish between substance dependent groups and non-dependent controls. In line with our theoretical predictions, the four-factor solution provided a very good fit to the Bulgarian data, indicating that the SURPS performs well in this translated measure. Factor loadings from the CFA were all above 0.30, with the exception of item 22 from the impulsivity subscale (“I feel I have to be manipulative to get what I want”), whose factor loading was 0.26. Conceptually, this item appears more closely related to psychopathy than to impulsivity *per se*. The SURPS also correlated well with theoretically related measures and was a very good predictor of associated outcomes, such as SUDs, indicating that the Bulgarian version of the scale has good concurrent and predictive validity.

Though the SURPS has demonstrated good psychometric properties with non-substance dependent adolescent and college student samples, its validity and clinical utility with substance dependent community samples remained unexplored. To our knowledge, this was the first study investigating the properties of the SURPS with non-treatment seeking community drug users with a history of substance dependence. In regard to the ability of the SURPS to distinguish between groups, we found significant group differences on the two externalizing SURPS factors (Impulsivity and Sensation Seeking), where the three substance dependent groups scored higher than controls. Contrary to expectations, we found no group differences on internalizing traits such as Hopelessness and Anxiety Sensitivity, which raises questions about the utility of these traits with substance dependent samples. The lack of association with internalizing traits may also be related to the protracted abstinence stage of the addiction cycle, which characterized the majority of our substance dependent participants. However, this hypothesis contradicts current mechanistic models of addiction (Koob and Volkow, 2010), where the withdrawal/negative affect stage of addiction is associated with negative reinforcement mechanisms and recruitment of brain stress systems related to internalizing traits and symptoms, such as depression and anxiety (Koob et al., 2014). These symptoms may be more prominent soon after discontinuation of drug use and may partially recover with longer periods of abstinence. In general, the protracted abstinence stage of the addiction cycle is relatively understudied and not well-understood. There is a need for systematic studies examining recovery of function with increasing length of abstinence in users of different classes of drugs.

One surprising finding was that all SURPS subscales except for the SURPS-SS were significantly associated with alexithymia, a personality trait characterized by difficulty identifying, differentiating, and expressing emotions, which is highly prevalent among substance dependent individuals (Morie et al., 2016). Alexithymia is proposed to be a coping strategy for dealing with negative emotions (Bilotta et al., 2016), reflecting deficits in cognitive processing (Fullam and Dolan, 2006) or in affect regulation (Lander et al., 2013) and empathy (Grynberg et al., 2010). Consistent with our findings, alexithymia has previously been associated with impulsivity (Shishido et al., 2013), anxiety sensitivity (Devine et al., 1999; Zahradnik et al., 2009), depression (Bamonti et al., 2010), and hopelessness (Izci et al., 2015). Of particular relevance to our findings is the recently proposed heuristic framework for Addictions Neuroclinical Assessment (Kwako et al., 2016), which considers alexithymia a key component of negative emotional states during the withdrawal/abstinence stage of addiction, characterizing the majority of our substance dependent participants, who were in protracted abstinence at the time of testing. Alexithymia may become potentially more important than anxiety sensitivity or hopelessness during the protracted abstinence phase of the addiction cycle. During the earlier stages of addiction, the cycling between active drug use and abstinence may sensitize substance users to the physiological symptoms of anxiety (i.e., anxiety sensitivity), and lead to feelings of hopelessness related to the inability to leave the cycle. The ability to remain abstinent may reduce hopelessness and anxiety sensitivity during the protracted abstinence stage of addiction. However, these negative affective states may be replaced by other dimensions of negative emotionality such as alexithymia and anhedonia, characterized by inability to express feelings or to experience positive emotions. Given that protracted abstinence is one of the least well-understood stages of the addiction cycle, this question needs more detailed investigation.

Even more surprising was the lack of associations between any of the SURPS subscales with heroin abuse or dependence. Given that the SURPS has been validated mainly with adolescents who primarily use alcohol, cannabis, and stimulants, the sensitivity of the scale in relation to heroin dependence needs more research. Heroin addiction has some unique characteristics that distinguish it from addictions to other classes of drugs, such as having the largest amount of drug-specific genetic variance and the least amount of shared genetic variance among illicit drugs (Tsuang et al., 1998). It is also associated with specific genetic susceptibility to opioid use vs. to drug use in general (Clark et al., 2016) and with more “severe” neurobiological changes and complications, to which the SURPS may be relatively insensitive. Further, our heroin dependent participants were characterized by longer duration of abstinence relative to amphetamine and polysubstance users. Therefore, the lack of associations between heroin dependence and the SURPS subscales could also be due to neuroplasticity and recovery of function in brain circuits affected by drugs of abuse.

All SURPS subscales except for the SURPS-SS were also significantly associated with ASPD, while the SURPS-IMP and SURPS-H were additionally significantly associated with CD. Consistent with our findings, there is evidence that disruptive behavioral disorders and mood disorders are often comorbid with SUDs (Roberts et al., 2007). The IMP subscale was also associated with several measures of psychopathy, SUDs, and childhood symptoms of ADHD, demonstrating that they may share the same psychopathological symptoms. Research shows that the covariance between SUDs and different dimensions of antisocial behavior could be modeled by a single underlying externalizing factor that is influenced by genetic risk (Kendler et al., 2003; Krueger et al., 2007; Patrick, 2008; Castellanos-Ryan and Conrod, 2011). However, the externalizing spectrum is highly correlated with the internalizing spectrum, which has been shown to be due to common genetic and environmental risk factors (Cosgrove et al., 2011).

The observed moderate correlation of AS with trait anxiety is in line with previous research demonstrating that AS and trait anxiety are distinct constructs (McNally, 1989). AS is currently viewed as being both an independent construct and a lower-order factor of trait anxiety (McWilliams and Cox, 2001). Our findings are consistent with the role of AS as a predictor of SUDs and related behaviors (Comeau et al., 2001; Novak et al., 2003), as shown by the significant associations between the AS subscale and nicotine dependence, number of symptoms of cannabis abuse, amphetamine dependence, antisocial personality disorder, and years of heroin use.

## Limitations

There are some limitations of our study that need to be noted. First, cultural differences between Bulgarian and North American/Western European populations limit the generalizability of our findings, though one of our primary goals was to validate the Bulgarian version of the SURPS. Second, our small sample size precluded investigations of potential sex differences in SURPS risk profiles and their relations to addiction to different classes of drugs. Third, we used DSM-IV abuse and dependence criteria. The use of DSM-5 might show slightly different results, since some of the criteria have changed. Fourth, some of the instruments were self-report measures (including some reverse-keyed items), which may lead to significant variations due to subjective and cultural factors.

## Conclusion

The Bulgarian version of the SURPS replicated the original factor structure of the scale and demonstrated acceptable to good reliability and validity. Accordingly, it is a useful tool for conducting research in SUDs and substance related disorders in Bulgaria. It also appears to be useful for assessing personality risk for SUD, which could be targeted by tailored personality-based interventions.

## Author contributions

SM, EL, and JV conceived the study. EL performed the statistical analyses, drafted the analysis and results sections, and led the revisions. SM drafted the introduction and discussion sections and assisted with the revisions. NG consulted on the statistical analyses. EP, GV, KB, and DN collected and managed the data. JV supervised the findings of this work, drafted portions of the manuscript, and oversaw the revisions. All authors discussed the results and contributed to the final manuscript.

### Conflict of interest statement

GV has ownership interests in the Bulgarian Addictions Institute, where data collection took place. The remaining authors declare that the research was conducted in the absence of any commercial or financial relationships that could be construed as a potential conflict of interest
